# Multiple Calcium Export Exchangers and Pumps Are a Prominent Feature of Enamel Organ Cells

**DOI:** 10.3389/fphys.2017.00336

**Published:** 2017-05-23

**Authors:** Sarah Y. T. Robertson, Xin Wen, Kaifeng Yin, Junjun Chen, Charles E. Smith, Michael L. Paine

**Affiliations:** ^1^Center for Craniofacial Molecular Biology, Herman Ostrow School of Dentistry, University of Southern CaliforniaLos Angeles, CA, United States; ^2^Department of Oral Medicine, Shanghai Ninth People's Hospital, Shanghai Jiao Tong University School of MedicineShanghai, China; ^3^Shanghai Key Laboratory of Tumor Microenvironment and Inflammation, Department of Biochemistry and Molecular Cell Biology, Shanghai Jiao Tong University School of MedicineShanghai, China; ^4^Department of Anatomy and Cell Biology, Faculty of Medicine, McGill UniversityMontreal, QC, Canada

**Keywords:** amelogenesis, biomineralization, calcium channels, calcium exchangers, calcium pumps

## Abstract

Calcium export is a key function for the enamel organ during all stages of amelogenesis. Expression of a number of ATPase calcium transporting, plasma membrane genes (ATP2B1-4/PMCA1-4), solute carrier SLC8A genes (sodium/calcium exchanger or NCX1-3), and SLC24A gene family members (sodium/potassium/calcium exchanger or NCKX1-6) have been investigated in the developing enamel organ in earlier studies. This paper reviews the calcium export pathways that have been described and adds novel insights to the spatiotemporal expression patterns of PMCA1, PMCA4, and NCKX3 during amelogenesis. New data are presented to show the mRNA expression profiles for the four Atp2b1-4 gene family members (PMCA1-4) in secretory-stage and maturation-stage rat enamel organs. These data are compared to expression profiles for all Slc8a and Slc24a gene family members. PMCA1, PMCA4, and NCKX3 immunolocalization data is also presented. Gene expression profiles quantitated by real time PCR show that: (1) PMCA1, 3, and 4, and NCKX3 are most highly expressed during secretory-stage amelogenesis; (2) NCX1 and 3, and NCKX6 are expressed during secretory and maturation stages; (3) NCKX4 is most highly expressed during maturation-stage amelogenesis; and (4) expression levels of PMCA2, NCX2, NCKX1, NCKX2, and NCKX5 are negligible throughout amelogenesis. In the enamel organ PMCA1 localizes to the basolateral membrane of both secretory and maturation ameloblasts; PMCA4 expression is seen in the basolateral membrane of secretory and maturation ameloblasts, and also cells of the stratum intermedium and papillary layer; while NCKX3 expression is limited to Tomes' processes, and the apical membrane of maturation-stage ameloblasts. These new findings are discussed in the perspective of data already present in the literature, and highlight the multiplicity of calcium export systems in the enamel organ needed to regulate biomineralization.

## Introduction

Enamel is the hardest and most calcified tissue in mammals, and understanding enamel formation is crucial for developing strategies to repair or regenerate it (Smith, 1998; Hubbard, 2000; Lacruz et al., 2013). Amelogenesis, the process of enamel development, can be divided into the secretory and maturation stages with a brief pre-secretory stage before the secretory stage and a transition stage between the secretory and maturation stages. Epithelial-derived enamel-forming cells (ameloblasts) differentiate from the inner enamel epithelium (IEE) during the pre-secretory stage (Orrenius et al., 2015). These amelobasts are highly polarized with an apical end that faces the enamel area and a basal end that faces the blood circulation. During the secretory stage, ameloblasts migrate away from the dentin while synthesizing and secreting enamel matrix proteins (EMPs) such as amelogenin, ameloblastin, and enamelin into the enamel area from Tomes' processes at their apical ends. These EMPs serve as a scaffold for the orientation and elongation of enamel hydroxyapatite (Hap) crystals (Smith, 1998). Each enamel rod follows a single ameloblast's Tomes' process with the interrod following the border of the cell, giving enamel its characteristic rod-interrod pattern (Skobe, 2006; Hu et al., 2007). There is a massive shift in gene expression during the transition stage, when approximately 25% of ameloblasts undergo apoptosis, after which another 25% undergo apoptosis throughout the following stages of amelogenesis (Tsuchiya et al., 2009). During the maturation stage, the ameloblasts undergo cyclical changes between ruffle-ended (RA) and smooth-ended (SA) morphology (Smith, 1998; Lacruz et al., 2013). Maturation-stage ameloblasts become specialized for ion transport and resorptive activities, which includes the secretion of the protease KLK4 to aid in the degradation of EMPs that are subsequently removed through endocytosis (Smith, 1998; Lacruz et al., 2012a, 2013). The continuously growing incisor of mice makes it a good model for studying the chronological progression of amelogenesis. While general concepts of ion transport throughout amelogenesis have been well-studied and discussed elsewhere (Arquitt et al., 2002; Paine et al., 2007; Lyaruu et al., 2008; Bronckers et al., 2010, 2015; Josephsen et al., 2010; Yin et al., 2015), and in particular the transcellular calcium ion (Ca^2+^) transport (reviewed in Nurbaeva et al., 2015b), in this paper we focus primarily on Ca^2+^ export.

### Overview—calcium transport

In general high intracellular concentrations of calcium (Ca^2+^) catalyze cell death signaling cascades, so cells maintain a gradient of ~10^−3^ M Ca^2+^ concentration outside the cell, in the mitochondria, and in the endoplasmic reticulum (ER) where Ca^2+^ is stored; while in the cytoplasm, the concentration is ~10^−7^ M (Brini and Carafoli, 2011). The plasma membrane contains a variety of Ca^2+^ channels that transiently open to allow Ca^2+^ influx in response to plasma membrane voltage changes, ligand-receptor interaction, or emptying Ca^2+^ stores of the ER and mitochondria (Brini and Carafoli, 2011). Calcium is removed from the cytoplasm through a number of mechanisms including the SERCA pump that replenishes ER stores, the mitochondrial Ca^2+^ uniporter, that replenishes mitochondrial stores, the plasma membrane low-affinity high capacity Na^+^/Ca^2+^ exchanger proteins (NCX), the Na^+^/Ca^2+^ K^+^ exchanger proteins (NCKX), and the high-affinity low-capacity plasma membrane Ca^2+^-ATPase (PMCA) pump proteins (Berridge et al., 2003; Brini and Carafoli, 2011; Hu et al., 2012; Bronckers et al., 2015).

Calcium (Ca^2+^) transport is crucial to understand the process of amelogenesis because not only is unbound Ca^2+^ a major component of hydroxyapatite (Hap), Ca^2+^ can also act as a major signaling molecule capable of regulating cell processes in eukaryotic cells such as cell division, cell attachment, motility, survival, differentiation as well as gene expression (Hubbard, 1996; Blair et al., 2011).

### Calcium extrusion

The SLC8A (sodium/calcium exchangers or NCX), SLC24A (potassium-dependent sodium/calcium exchangers or NCKX), and ATP2B (ATPase plasma membrane Ca^2+^ transporting pumps or PMCA pumps) gene families of Ca^2+^ transporters mediate Ca^2+^ extrusion in most cell types (Brini and Carafoli, 2011), and proteins from all three of these families have been reported in enamel organ cells (Sasaki and Garant, 1986c; Borke et al., 1995; Zaki et al., 1996; Okumura et al., 2010; Hu et al., 2012; Wang et al., 2014). The SLC8A gene family has 3 members (NCX1-3) and all have a generally accepted stoichiometry of the extrusion of 1 Ca^2+^ in exchange for the intrusion of 3 Na^+^ (Brini and Carafoli, 2011), while the SLC24A gene family has 5 members (NCKX1-5) and extrudes 1 Ca^2+^ and 1 K^+^ in exchange for 4 Na^+^, typically against the Ca^2+^ gradient; however, the directionality both NCX and NCKX exchangers can be reversed depending on the Na^+^ and Ca^2+^ gradients (Jalloul et al., 2016b; Zhekova et al., 2016). SLC8A and SLC24A gene families are electrogenic because there is a translocation of net charge across the plasma membrane, have a low Ca^2+^ affinity, and are capable of transporting Ca^2+^ in bulk rapidly across the plasma membrane (Brini, 2009). They are reversible but in ameloblasts they likely operate in extruding Ca^2+^ from the cytoplasm facilitated by transport of Na^+^ and K^+^ down their gradients (Brini and Carafoli, 2011; Hu et al., 2012). PMCA pumps/proteins have 4 members (PMCA1-4, coded by genes ATP2B1-4) and are part of a larger family of genes, called P-type primary ion transport ATPases, that catalyze the auto-phosphorylation of a conserved aspartyl residue within the pump from ATP (Palmgren and Nissen, 2011).

### Calcium extrusion—SLC8A and SLC24A gene products

The SLC8A and SLC24A families are primarily expressed in excitable tissues such as muscle and heart, as their rapid bulk transport of Ca^2+^ is important in, for example, muscle and heart contraction (Brini and Carafoli, 2011). The SLC8A/NCX and SLC24A/NCKX transporters are Na^+^/Ca^+^ exchangers and can be either K^+^-dependent (NCKX) or K^+^-independent (NCX) (Shumilina et al., 2010).

NCX1 is expressed in heart, brain, bladder, kidney, and cells of the enamel organ; NCX2 is expressed in brain and skeletal muscle; and NCX3 is expressed in brain, skeletal muscle and cells of the enamel organ (Lytton, 2007; Okumura et al., 2010; Lacruz et al., 2012b; Sharma and O'halloran, 2014). Okumura et al. demonstrated NCX1 and NCX3 expression at the apical pole of both secretory and maturation ameloblasts, and expression of NCX1 was also observed in cells of the stratum intermedium and papillary layer (Okumura et al., 2010). In addition, protein levels of NCX1 and NCX3 throughout amelogenesis remained relatively unchanged (Okumura et al., 2010). Using real-time PCR, Lacruz et al. confirmed that the mRNA levels of both NCX1 and NCX3 did not significantly change from secretory- to maturation-stage enamel organ cells (Lacruz et al., 2012b).

NCKX1 is expressed primarily in retinal rod photoreceptors and platelets (Schnetkamp, 2004; Lytton, 2007). NCKX2 is expressed in cone photoreceptors and is involved in mouse motor learning and memory (Schnetkamp, 2004; Lee et al., 2009, 2013), and NCKX3 is expressed in the brain and the kidneys (Schnetkamp, 2004; Lee et al., 2009) though it is expressed in the kidneys at higher levels in female mice than in male mice (Lee et al., 2009). NCKX3 is also highly expressed in the human endometrium during the menstrual cycle, where its expression is partially regulated by the steroid hormone 17β-estradiol (Yang et al., 2011). NCKX4 is expressed in olfactory neurons (Stephan et al., 2011), and also in the maturation-stage ameloblasts (Hu et al., 2012). NCKX5 is expressed in skin melanocytes, retinal epithelium, and brain (Schnetkamp, 2004; Lytton, 2007; Sharma and O'halloran, 2014; Jalloul et al., 2016a,b). NCKX6/NCLX was originally considered a member of the NCKX family but is now considered part of the Ca^2+^ cation (CCX) exchanger branch (Cai and Lytton, 2004; Sharma and O'halloran, 2014) as a mitochondrial membrane Ca^2+^, Li^+^/Na^+^ exchanger with a wide tissue distribution (Schnetkamp, 2004; Lytton, 2007; Sharma and O'halloran, 2014).

### Calcium extrusion—ATP2B gene products

The ATPase plasma membrane Ca^2+^ transporting (or PMCA) gene family is postulated to be involved in Ca^2+^ homeostasis, as it has a high affinity for Ca^2+^ but cannot transport Ca^2+^ as rapidly as either the NCX or NCKX transporters (Brini and Carafoli, 2011). The PMCA family is part of the superfamily of P-type ATPase pumps that form a stable phosphorylated intermediate as it hydrolyzes one molecule of ATP for each Ca^2+^ transported (Strehler and Zacharias, 2001; Cai and Lytton, 2004). The phosphorylated enzyme intermediate of the P-type ATPases, which include the SERCA family of transporters in the ER membrane (Giacomello et al., 2013), occurs between γ-phosphate of a hydrolyzed ATP with a D-residue in a highly conserved region of the ATPase pump (Brini and Carafoli, 2011).

PMCA1 is expressed in most tissues throughout development. Its expression is highest in the nervous system, heart, skeletal muscle, and intestine (Zacharias and Kappen, 1999), and regulated by growth factors such as glucocorticoids and Vitamin D (Zacharias and Kappen, 1999; Giacomello et al., 2013). PMCA2 is expressed mainly in the brain, heart, mammary glands and ear, and decreased expression of PMCA2 causes increased apoptosis in breast cancer cells (Curry et al., 2012; Giacomello et al., 2013). PMCA3 has the highest calmodulin affinity and is detected primarily in the brain and skeletal muscles (Krebs, 2009; Giacomello et al., 2013). PMCA4 is involved in the fertilization process and cardiac function, and has been found to associate with lipid rafts, which often function to aggregate protein complexes important in signaling pathways (Giacomello et al., 2013). It has been suggested that PMCA4 is more involved in cell-specific Ca^2+^ signaling than as a pump for bulk Ca^2+^ export (Strehler, 2013; Brini et al., 2017). PMCA4 interacts with nitric oxide synthase and with calcineurin, which regulates NFAT signaling (Brini, 2009; Kim et al., 2012; Strehler, 2013).

PMCA1 and PMCA4 are important in osteoclast differentiation, maturity, and survival, and *Atp2b1*^+/−^ and *Atp2b4*^−/−^ mice have decreased bone density due to an increased number of mature osteoclasts and increased osteoclast apoptosis (Kim et al., 2012). The PMCA family members can also influence IP_3_-mediated calcium signaling by binding to phosphatidylinositol-4,5-bisphosphate (PIP_2_) on the plasma membrane as well as removing Ca^2+^ necessary for phospholipase C (PLC) activity, which prevents cleaving by PLC and thereby prevents Ca^2+^ release from the ER (Penniston et al., 2014). Altered PMCA expression is a characteristic of many cancers (Curry et al., 2011) and many other human diseases (Brini et al., 2013), but the diversity in isoforms and splicing and lack of specificity of small molecules to target PMCAs present challenges in therapeutic agent development (Strehler, 2013).

### Summary—calcium export exchangers and pumps in amelogenesis

There now are a number of reports that show expression and localization data for NCX1 and NCX3 (Okumura et al., 2010; Lacruz et al., 2012b), and NCKX4 (Hu et al., 2012; Wang et al., 2014) in the enamel organ. Reports on PMCA expression and activities throughout amelogenesis are scant (Sasaki and Garant, 1986c; Borke et al., 1995; Zaki et al., 1996), and to the authors' knowledge, the only investigations into the role of PMCA proteins in amelogenesis date back decades. Data presented here better defines the mRNA profiles of all SLC8A, SLC24A, and ATP2B gene family members, and adds additional insight into the protein and spatiotemporal expression profiles of NCKX3, PMCA1, and PMCA4.

### Calcium export exchangers and pumps and disease

A number of the ATP2B, SLC8A, and SLC24A gene family members are linked to mammalian disease, but notably mutations to *SLC24A4* are associated with non-syndromic amelogenesis imperfecta (AI) (Parry et al., 2013; Seymen et al., 2014; Wang et al., 2014; Herzog et al., 2015). A comprehensive list of the PMCA, NCX, and NCKX pumps and exchangers, their links to human pathologies, and mouse models of each gene is found in Table 1.

**Table 1:** **Pathologies associated with genes ATP2B1-4, SLC8A1-3 and SLC24A1-6**.

**Gene symbol**	**Protein name**	**Predominant substrates**	**Link to human disease**	**Animal models**	**References**
ATP2B1	PMCA1	Ca^2+^		Embryonic lethal	Okunade et al., 2004
ATP2B2	PMCA2	Ca^2+^		Vestibular/motor imbalance, Deafness	Street et al., 1998; Bortolozzi et al., 2010
ATP2B3	PMCA3	Ca^2+^	Spinocerebellar ataxia		Bertini et al., 2000; Zanni et al., 2012
ATP2B4	PMCA4	Ca^2+^	Familial spastic paraplegia	No overt phenotype in Atp2b4 null mice, Male mice are infertile	Okunade et al., 2004; Ho et al., 2015
SLC8A1	NCX1	Na^+^, Ca^2+^		Embryonic lethal	Wakimoto et al., 2000
SLC8A2	NCX2	Na^+^, Ca2^+^			
SLC8A3	NCX3	Na^+^, Ca^2+^		Skeletal muscle fiber necrosis, Defective neuromuscular transmission	Sokolow et al., 2004
SLC8B1^*^	NCLX	Na^+^, Li^+^, Ca^2+^			Khananshvili, 2013
SLC24A1	NCKX1	Na^+^, Ca^2+^, K^+^	Congenital stationary night blindness	Night blindness	Riazuddin et al., 2010; Vinberg et al., 2015
SLC24A2	NCKX2	Na^+^, Ca^2+^, K^+^			
SLC24A3	NCKX3	Na^+^, Ca^2+^, K^+^			
SLC24A4	NCKX4	Na^+^, Ca^2+^, K^+^	Amelogenesis imperfecta	Amelogenesis imperfecta	Parry et al., 2013; Seymen et al., 2014; Wang et al., 2014; Herzog et al., 2015
SLC24A5	NCKX5	Na^+^, Ca^2+^, K^+^	Hypopigmentation, Oculocutaneous albinism		Mondal et al., 2012; Wei et al., 2013
SLC24A6	NCKX6	Na^+^, Ca^2+^, K^+^			

*Note that SLC8B1 is found in the mitochrodria of mammalian skeletal and heart muscle, neurons and a few other cell types*.

## Materials and methods

### Animals

All vertebrate animal manipulation was carried out in accordance with Institutional and Federal guidelines. The animal protocols were approved by the Institutional Animal Care and Use Committee at the University of Southern California (Protocol #20461).

### Quantitative PCR analysis

Secretory-stage and maturation-stage enamel organ cells from mandibular incisors of 4-week old Wistar Hanover rats were collected as previously described (Lacruz et al., 2012b; Wen et al., 2014), and RNA extraction was performed using a QIAshredder, an RNeasy Protect Mini Kit, and DNase I solution from Qiagen (Valencia, CA, USA). Reverse transcription and real-time PCR were performed using the iScript cDNA Synthesis kit and SYBR Green Supermix from BioRad, respectively. Real-time PCR was performed on the CFX96 system (BioRad Laboratories, Hercules, CA, USA) in 10 μl volumes with a final primer concentration of 100 nm, for 40 cycles at 95°C for 10 s and 58°C for 45 s. Six independent real-time PCR analyses were conducted using samples from a total of 6 rats, 3 males, and 3 females, for each gene of interest (primers are listed in Table 2), and for both stages of amelogenesis. The male and female data were analyzed separately and no significant differences were noted between the sexes, so the data presented in the graph were generated from all 6 animals (*n* = 6). Rat enamel organ is preferred to mouse enamel organ for real-time PCR and western blot studies because separating secretory and maturation stage from adult mouse incisors is technically difficult and yields less RNA and protein per animal.

**Table 2:** **Primers used for real-time PCR**.

**Symbol**	**Accession**	**Size**	**Region**	**Forward**	**Reverse**	**Temp**
Atp2b1	NM_053311	219	1,387–1,605	AAAGCAGGTCTGCTGATGTC	GACGGAGTAAGCCAGTGAGA	58
Atp2b2	NM_012508	168	4,129–4,296	GAGACGTCGCTTTAGCTGAG	AAAGGGTCTGTGTGTGGAAA	58
Atp2b3	NM_133288	207	4,073–4,279	GCTCCATGACGTAACCAATC	GCGGAATATTGTGGGTGTAG	58
Atp2b4	NM_001005871	184	3,698–3,881	AATCCAAGAACCAGGTCTCC	ACGGCATTGTTATTCGTGTT	58
Slc8a1	NM_019268	150	2,430–2,579	CCTGCTTCATTGTCTCCATC	CAAATGTGTCTGGCACTGAG	58
Slc8a2	NM_078619	166	377–542	AAACGGTGTCCAACCTTACA	ACACACACAGCAATGACCAC	58
Slc8a3	NM_078620	206	4,310–4,515	TGGTGGAAGCCATTCTATGT	AATATGGCCCACTCCCTTAG	58
Slc24a1	NM_004727	155	3,238–3,392	TTCCTGACCTCATCACCAGT	TGGAACTGGCTGTAATCCAT	58
Slc24a2	NM_031743	214	1,280–1,493	GGGAGGTTCAGAGAAAAAGC	CGATGCTGTGAGAGAGGTTT	58
Slc24a3	NM_053505	150	2,941–3,090	TGACATGTGCTCTTGTTGCT	AATTGGGACTTCATTGACGA	58
Slc24a4	NM_001108051	235	401–635	AAAGTTGATGGCACCGATAA	AGGGATGGGACAAAGAAGTC	58
Slc24a5	NM_001107769	161	371–531	AACATGGTTTCAACGCTCTC	CACAGCAGCAGGACATACAG	58
Slc24a6	NM_001017488	160	613–772	TTCTCAGACCCTCGTACTGC	ACACGGCCACCATATAGAAA	58
Enam	NM_001106001	169	1,139–1,307	ATGCTGGGAACAATCCTACA	GTGGTTTGCCATTGTCTTTC	58
Odam	NM_001044274	206	658–863	TTGACAGCTTTGTAGGCACA	GACCTTCTGTTCTGGAAGCAG	58
Actb	NM_031144	272	559–830	CACACTGTGCCCATCTATGA	CCGATAGTGATGACCTGACC	58

### Western blot analysis

Secretory (S) and maturation (M) enamel organ cells from mandibular incisors of 4-week old Wistar Hanover rats were collected. Brain (B) and heart (H) tissues were also collected as control tissues. Total protein extraction was performed with RIPA buffer (1% Nonidet P-40, 0.1% SDS, 0.5% deoxycholic acid, 150 mm NaCl, 50 mm Tris, pH 8.0) and protease inhibitor cocktail, complete mini (Roche Applied Sciences, Indianapolis, IN, USA). Samples were homogenized manually with a pestle six times, then sonicated with a BRANSON digital sonifier Model 450 (All-Spec Industries, Wilmington, NC, USA; 10% intensity, 10 s on and 10 s off). Samples were then cleared by centrifugation (15,000 g, 15 min, 4°C). Proteins were quantified using the bicinchoninic acid (BCA) assay (Pierce, Rockford, IL, USA) and equal quantities were loaded (15 μg per lane) onto 4–12% SDS–PAGE resolving gels. Protein was transferred to a PVDF membrane, then blocked with 5% milk in TBST. Antibodies against PMCA1 (AbCam, Cambridge, MA, USA; catalog #ab190355), PMCA2 (ab3529), PMCA3 (ab3530), PMCA4 (ab2783), NCKX3 (St. John's Laboratory, London, UK, catalog #STJ94358), GAPDH (Santa Cruz Biotechnology, Santa Cruz, CA, USA, catalog #sc-32233), amelogenin (ThermoFisher Scientific, catalog #PA5-31286), and cardiac muscle actin (ACTC1) (GeneTex Inc., Irvine, CA, catalog #GTX101876) were used at dilutions of 1:500, 1:2,000, 1:300, 1:5,000, 1:500, 1:500, 1:3,000, and 1:500, respectively in 5% milk in TBST. Secondary antibody for PMCA1-4 from Cell Signaling (Danvers, MA, USA; catalog #7074 and #7076) was applied at a dilution of 1:10,000. Secondary antibodies for NCKX3, amelogenin, and ACTC1 from Santa Cruz Biotechnology (Santa Cruz, CA, USA; catalog #sc-2004 and sc-2418) were applied at a dilution of 1:7500. Pierce ECL Plus Western Blotting Substrate (Thermo Scientific, Rockford, IL, USA; catalog #32132) was used as the detection system for all antibodies. TBST (tris-buffered saline with.1% Tween-20) was used as a wash buffer.

### Immunofluorescence

Mandibular incisors were dissected from 9-day-old wild type mice and placed in 4% paraformaldehyde in PBS overnight. Mouse incisors were preferred to rat incisors in the immunofluorescence studies because mouse incisors decalcify more rapidly and all stages of amelogenesis are visible in one sagittal section. The incisors were then washed in PBS and decalcified in 10% EDTA in PBS pH 7.4 for 4 weeks at 4°C. The sample was embedded in paraffin and 4 μm sections were cut with a microtome. The sections were deparaffinized and rehydrated. The primary antibodies for PMCA1 and PMCA4 (AbCam, Cambridge, MA, USA; catalog #ab3528 and #ab2783, respectively) were used at dilutions of 1:40 and 1:200 in 1% BSA in PBS, respectively. The primary antibody for NCKX3 (Santa Cruz Biotechnology, Santa Cruz, CA, USA; catalog #sc-50129) was used at a dilution of 1:50. The secondary antibodies (Vector Laboratories, Burlingame, CA, catalog #DI-1088, DI-2488, DI-2594, DI-3094) were used at a dilution of 1:300 in 1% BSA in PBST. Sections were mounted with mounting medium with DAPI (Vector Laboratories, Burlingame, CA, catalog #H-1200) and imaged on a Leica TCS SP8 confocal microscope (Leica Biosystems). PBST (0.1% Tween-20) was used as a wash buffer for the experiments. Negative control sections, using secondary antibody only, under identical conditions, were included and showed negligible auto fluorescence—see Supplemental Figure 1.

## Results

### Messenger RNA expression profiles

**Figure 1:**
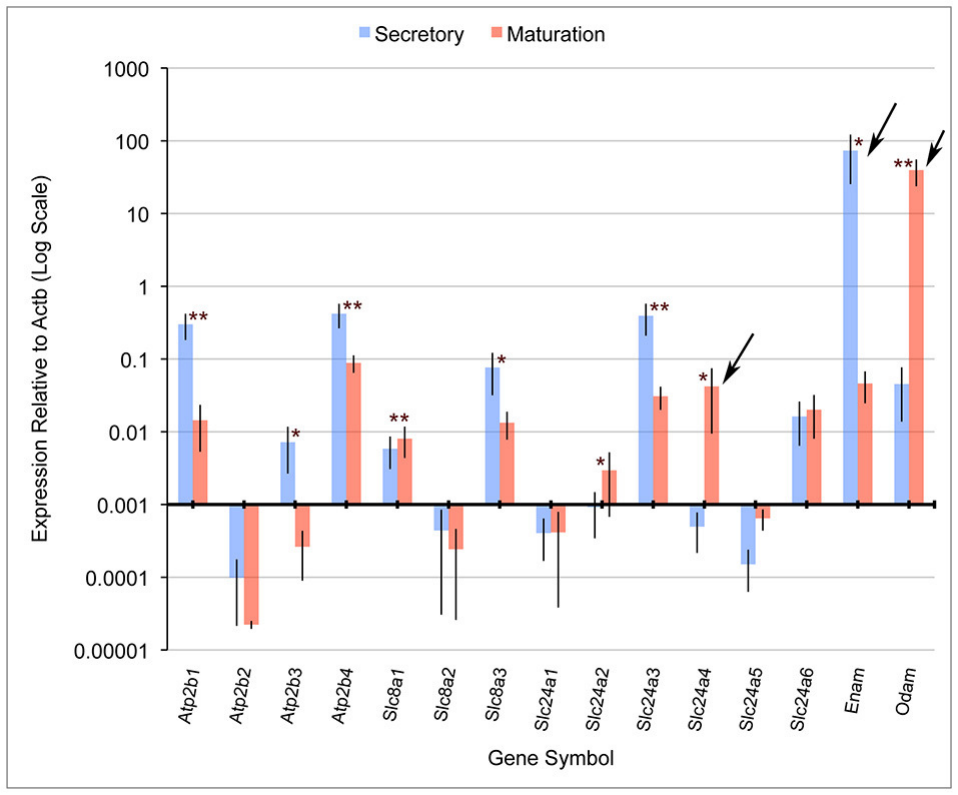
**Real-time PCR for rat Atp2b, Slc8a, and Slc24a gene family members**. Atp2b1, Atp2b3, Atp2b4, Slc8a3, and Slc24a3 have significantly higher expression in secretory stage than maturation stage, while Slc8a1, Slc24a2, and Slc24a4 were significantly more highly expressed in maturation stage compared to secretory stage. β-actin (Actb) served as a normalizing control, and enamelin (Enam) and Odam as control transcripts that were significantly down-regulated and up-regulated, respectively (as expected), during maturation-stage amelogenesis. Slc24a4, Enam and Odam (arrows) have all been linked to non-syndromic cases or amelogenesis imperfecta. The x-axis is placed at the 0.001 expression level relative to Actb, and below this “cut-off” figure is arbitrarily considered non-significant. The Student's *t*-test (paired two-tail) was used to compare the expression of each gene between the secretory and maturation stages (^*^p < 0.05, and ^**^p < 0.01). Standard deviations are also included.

Quantitative PCR (qPCR) comparing mRNA expression levels in secretory and maturation enamel organ cells for all PMCA (Atp2b), NCX (Slc8) and NCKX (Slc24) gene family members indicate that: (1) PMCA1 (*Atp2b1*), 3 (*Atp2b3*), and 4 (*Atp2b4*), and NCKX3 (*Slc24a3*) expression is highest during secretory-stage amelogenesis; (2) NCX1 (*Slc8a1*) and 3 (*Slc8a3*), and NCKX6 (*Slc24a6*) were expressed during secretory and maturation stages; and (3) NCKX4 (*Slc24a4*) is most highly expressed during maturation-stage amelogenesis (Figure 1). The expression levels of PMCA2 (*Atp2b2*), NCX2 (*Slc8a2*), NCKX1 (*Slc24a1*), NCKX2 (*Slc24a2*), and NCKX5 (*Slc24a5*) are negligible throughout amelogenesis (Figure 1). These data for NCX (Slc8) and NCKX (Slc24) gene family members are consistant with previously published gene expression data (Okumura et al., 2010; Hu et al., 2012), and add novel information suggesting that PMCA1, PMCA4, and to a lesser extent PMCA3 (which is expressed in secretory enamel organ cells at a level an order of magnitude lower than seen for PMCA1 and PMCA4), play an important role in secretory-stage amelogenesis.

### Western blot analysis confirms expression of PMCA proteins in enamel organ cells

**Figure 2:**
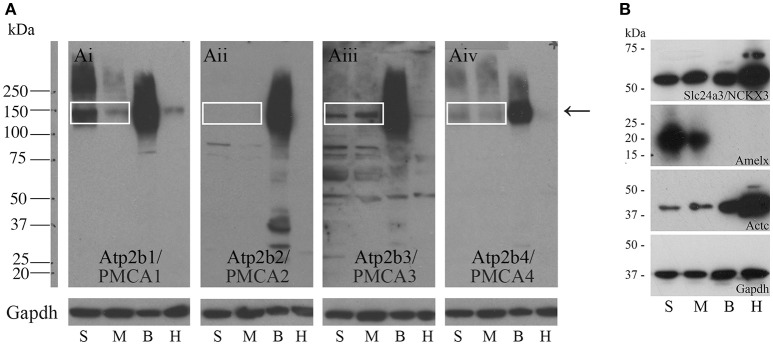
**Western blot analyses of PMCA1-4, and NCKX3 in secretory-stage and maturation-stage rat enamel organs. (A)** Western blot analysis for PMCA1 **(Ai)**, PMCA2 **(Aii)**, PMCA3 **(Aiii)** and PMCA4 **(Aiv)**. Samples are secretory-stage enamel organ cells (S), maturation-stage enamel organ cells (M), brain tissue (B) and heart tissue (H). Brain and heart samples are shown for comparison, as all PMCAs are highly expressed in brain and at lower levels in the heart (Brini and Carafoli, 2011; Brini et al., 2017). Molecular weight markers are indicated at left. The expected molecular weights for PMCA1, PMCA2, PMCA3, and PMCA4 are ~130, 133, 123, and 129 kDa, respectively. The bands are seen for PMCA1, PMCA3, and PMCA4 (boxed and arrow). No expression of PMCA2 is evident. GAPDH is used here as a loading control. **(B)** Western blot analysis of NCKX3. The expected molecular weight for NCKX3 is ~60 kDa. NCKX3 is expressed in all 4 tissue samples tested, with similar expression noted in both secretory-stage and maturation-stage enamel organ cells, and brain tissue. Relatively higher levels of NCKX3 expression can be appreciated in heart tissue. Amelx and Actc are used as controls as expression is highest in secretory-stage ameloblasts (Lacruz et al., 2012a,b) and heart tissue (Hamada et al., 1982) respectively. GAPDH is used here as a loading control.

Western blot data indicate that PMCA1 and PMCA4 are more highly expressed in secretory stage than in maturation stage, and PMCA2 is not expressed at any appreciable level in amelogenesis, consistent with the qPCR data (Figures 2Ai,Aiv,Aii respectively). Contrary to the qPCR data, PMCA3 and NCKX3 appear to be expressed at similar levels during both secretory stage and maturation stage (Figures 2Aiii,B respectively). Rat brain and heart protein samples were used as control tissues and analyzed with the secretory- and maturation-stage protein samples. The expected molecular weights for PMCA1-4 are ~130, 133, 123, and 129 kDa respectively, and relate to the single bands seen at approximately the 150 kDa molecular weight mark (as indicated by an arrow, Figure 2A). The expected molecular weight of NCKX3 is ~60 kDa (Figure 2B). Gapdh has been included as a loading control for all samples, and additional controls include Western analysis for both amelogenin (Amelx) and cardiac muscle alpha actin (Actc) (Figure 2B).

### PMCA1 and PMCA4 localization by immunofluorescence

**Figure 3:**
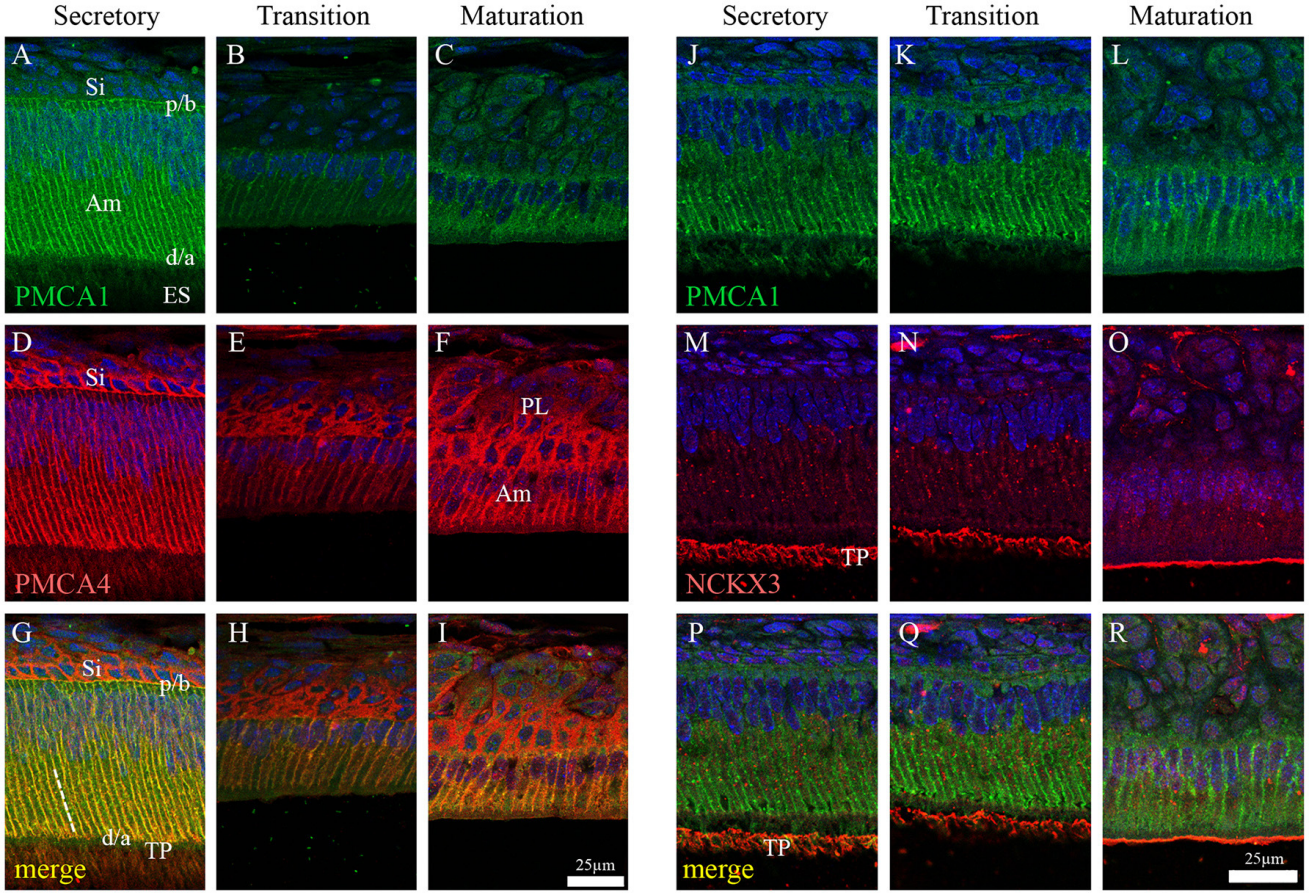
**Immunofluorescence analysis of PMCA1, PMCA4, and NCKX3 in 9-day-old mouse mandibular incisors**. Columns from left to right show secretory-stage **(A,D,G,J,M,P)**, transition-stage **(B,E,H,K,N,Q)** and maturation-stage **(C,F,I,L,O,R)** ameloblasts; while rows show immunoreactivity for PMCA1 (green; **A–C** and **J–L**), PMCA4 (red; **D–F**) and NCKX3 (red; **M–O**). Merged images are also shown for each column (**A,D** merged to **G**; **B,E** merged to **H**; **C,F** merged to **I**; **J,M** merged to **P**; **K,N** merged to **Q**; and **L** and **O** merged to **R**). Am, Ameloblasts; ES, enamel space; Si, stratum intermediu (TP; secretory ameloblasts only), Tomes' processes and (PL, maturation ameloblasts only), papillary layer. The proximal/basal poles (p/b) and distal/apical poles (d/a) of ameloblast cells are identified, as are the lateral membranes of ameloblasts (broken while line in **G**). Scale for **(A–I)** shown in **(I)**; and scale for **(J–R)** shown in **R**.

In the enamel organ, PMCA1 expression is seen primarily on the basolateral membrane of both secretory- and maturation-stage ameloblasts, with stronger signals seen in secretory ameloblasts (green; Figures 3A–C). These data complement both the qPCR (Figure 1) and Western blot data (Figure 2) on the spatiotemporal expression of PMCA1 in enamel organ cells. When compared to ameloblasts, a weaker signal of PMCA1 is seen in the stratum intermedium (Figure 3A) and papillary layer cells of the enamel organ (Figure 3B); as reflected by the orange color observed in the merged images (Figures 3G–I). In the enamel organ, PMCA4 expression is also seen on the basolateral membrane of secretory- and maturation-stage ameloblasts, and also cells of the stratum intermedium and papillary layer cells (red; Figures 3D–F). Similar to the PMCA1 data, these PMCA4 immunolocalization data complement the qPCR and Western blot data (Figures 1, 2). The co-localization of both PMCA1 and PMCA4 in polarized ameloblasts can be appreciated in the merged image (yellow; Figures 3G–I), while PMCA4 (but not PMCA1) is also expressed in the stratum intermedium and papillary layer cells of the enamel organ (red; Figures 3G–I).

### NCKX3 localization by immunofluorescence

NCKX3 expression is highest in the Tomes' processes (Figure 3M) and the apical membrane of transition- and maturation-stage ameloblasts (Figures 3N,O), while some minor ameloblast-specific intracellular granular immune-reaction is also apparent (Figures 3M–O). We compared the expression profile for NCKX3 (red; Figures 3M–O) to the expression profile of the control PMCA1 (green; Figures 3J–L). As can be appreciated from the images (Figures 3M–R), the expression profile for NCKX3 in the enamel organ is highest at the distal/apical pole, and this is distinct from the expression profiles seen for PMCA1 and PMCA4 where expression is seen on the lateral membranes of polarized ameloblasts (for both PMCA1 and PMCA4) and stratum intermedium and papillary layer cells (only PMCA4) (Figures 3A–L).

## Discussion

From data presented here and prior studies, it is possible to make the following generalizations. First, of the four unique genes coding the PMCAs, PMCA1, and PMCA4 are highly expressed on the basolateral membranes of polarized ameloblasts; and both are expressed during secretory- and maturation-stage amelogenesis. These data somewhat contradicts previously published data suggesting PMCA1 and PMCA4 are localized primarily to Tomes' processes of secretory ameloblasts (Sasaki and Garant, 1986c; Borke et al., 1995). These differences likely result from the different specificities of antibodies used to carry out these studies, and as noted previously, protein localization differences may also result from the different chemical and processing techniques used by the various laboratories (Takano, 1995). While expression of PMCA1 is primarily in the basolateral membrane of ameloblasts, there are also lower expression levels noted in the cells of stratum intermedium and papillary layer. Similarly, while expression of PMCA4 is seen in the basolateral membrane of ameloblasts, expression of PMCA4 is also recognized as a feature of the cells of the stratum intermedium and papillary layer cells. Of the three unique genes coding for the NCXs, NCX1, and NCX3 are highly expressed at the apical pole of both secretory- and maturation-stage ameloblasts (Okumura et al., 2010). Finally, of the six unique genes coding for NCKXs, NCKX3 (data reported here; Figures 1–3) and NCKX4 (Hu et al., 2012; Wang et al., 2014) are both highly expressed at the apical pole of polarized ameloblasts. A similar level of expression of NCKX3 is noted in both secretory- and maturation-stage ameloblasts (Figures 2, 3). While expression of NCKX4 is negligible in secretory-stage ameloblasts, it is highly expressed in maturation-stage ameloblasts (Hu et al., 2012; Wang et al., 2014). All six proteins expressed in ameloblasts (PMCA1, PMCA4, NCX1, NCX3, NCKX3, and NCKX4) export Ca^2+^ from the cytoplasm to the extracellular space, thus ameloblasts may be one of the more complicated epithelial cell types when it comes to understanding ion movements related to Ca^2+^ transport as they relate to a mineralizing dental enamel.

The data suggest that there are likely redundancies amongst similarly functioning proteins from these gene families. For example, from this list of six Ca^2+^ export proteins expressed in ameloblasts, only mutations to SLC24A4/NCKX4 have been linked to enamel pathologies (Parry et al., 2013; Seymen et al., 2014; Wang et al., 2014; Herzog et al., 2015). NCKX4 exports Ca^2+^ from the apical pole of maturation-stage ameloblasts at the developmental stage where enamel mineralization is at its greatest; thus, NCKX4 may play a greater role in enamel formation than either NCX1 or NCX3, which have expression localized to the apical pole throughout the entire process of amelogenesis. It is conceivable that if the function of either NCX1 or NCX3 is less than optimal, the other may compensate such that no overt enamel phenotype results. Future studies may be able to address whether NCX1 and NCX3 are equivalent in enamel formation.

Similar to NCX1 and NCX3 in ameloblasts, PMCA1 and PMCA4 or other calcium handling proteins may be able to overcome the effects of *Atp2b1* or *Atp2b4* mutations, or gene silencing. No human pathologies have yet been linked to *ATP2B1* mutations (as noted in the Online Mendelian Inheritance in Man; http://omim.org/entry/108731), however *Atp2b1*-null mice are embryonic lethal (Okunade et al., 2004). Only recently a case of familial spastic paraplegia has been linked to an *ATP2B4* mutation (Ho et al., 2015; http://omim.org/entry/108732), and while *Atp2b4-*null mice have no overt phenotype, the male mice are infertile due to reduced sperm motility (Okunade et al., 2004; Schuh et al., 2004; Kim et al., 2012). The similar expression profiles of PMCA1 and PMCA4 in ameloblasts (that being to the basolateral membrane) may suggest that loss of PMCA4 function in ameloblasts may be compensated by PMCA1, while the loss of PMCA1 function remains embryonic lethal. If this is correct, studying PMCA1 activities in *in vivo* enamel formation would, in the future, be limited to conditional knockout or heterozygote animal models. The localization of PMCA1 and PMCA4 on the ameloblast basolateral membrane may suggest that these Ca^2+^ pumps are unlikely to have a critical role in enamel mineralization; i.e., Ca^2+^ removed by the PMCA pumps may not directly be incorporated into the Hap mineral phase. Instead, the PMCA pumps may be indirectly involved in amelogenesis by maintaining ameloblast Ca^2+^ homeostasis; or being a part of ameloblast cell signaling pathways. As calcium is transported through the stratum intermedium and papillary layer to the ameloblasts and the enamel organ, the PMCA family may additionally be valuable in shuttling the calcium from circulation to the ameloblasts. The PMCA family can be involved in IP_3_-mediated calcium signaling (Penniston et al., 2014), and PMCA1 and PMCA4 are involved in RANKL signaling and regulate osteoclast differentiation (Kim et al., 2012). In cultured osteoclasts, the knockdown and/or silencing of both PMCA1 and PMCA4 increased protein expression of SERCA2 and TRPV5 (Kim et al., 2012). Indeed, the PMCA transporters may have evolved to fine-tune intracellular calcium concentration as they have higher calcium affinity and lower capability for bulk Ca^2+^ transport than the NCX/NCKX exchangers (Brini and Carafoli, 2011), and are therefore more likely to play a housekeeping role by removal of intracelullar Ca^2+^ during maturation stage enamel mineralization which may also prevent calcium overload and possibly ameloblasts apoptosis. The PMCA transporters have multiple known expressed isoforms in other tissues but this has not yet been studied in the enamel organ.

**Figure 4:**
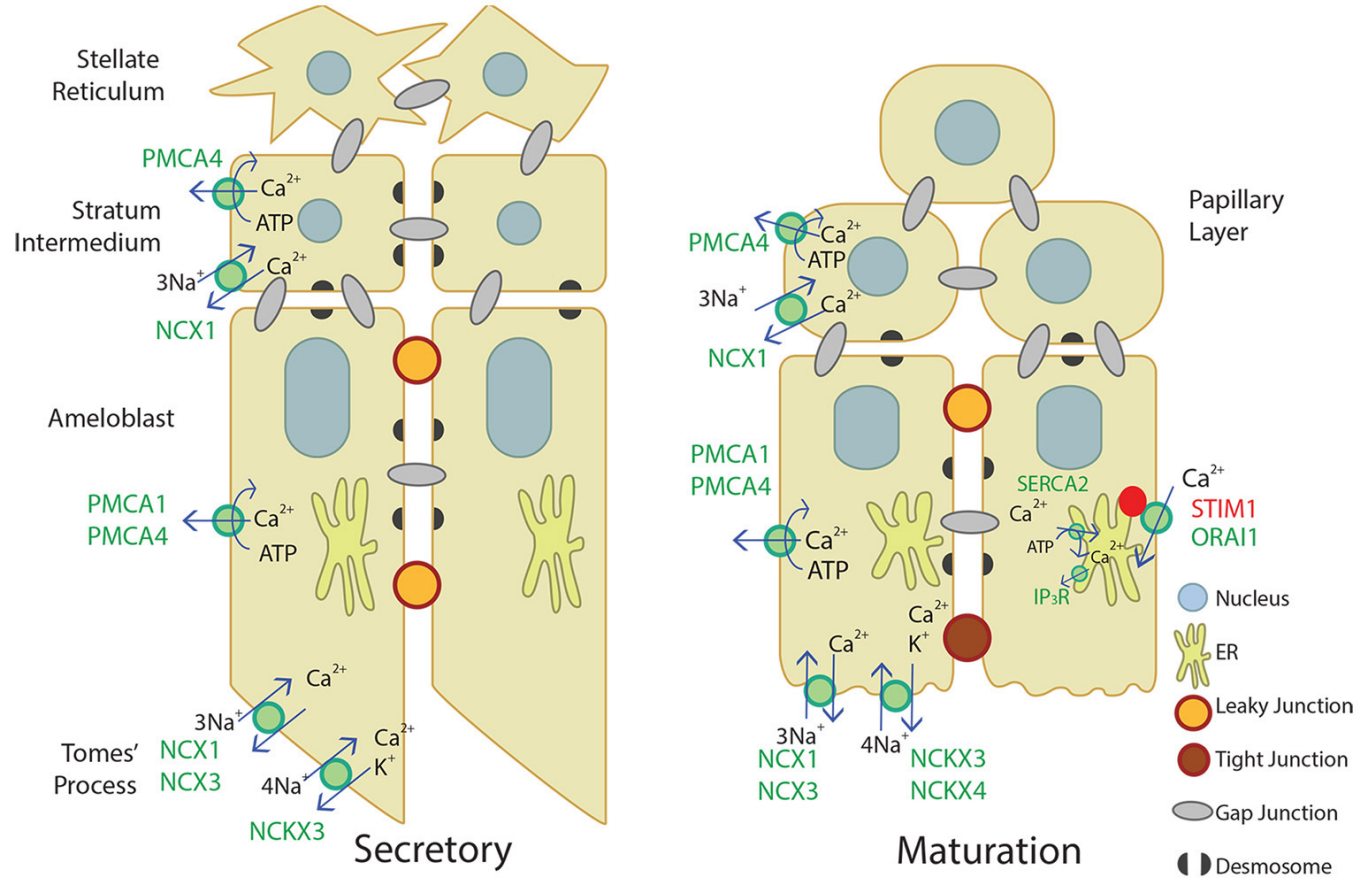
**Schematic of what is currently proposed for secretory- and maturation-stage transcellular calcium transport in amelogenesis**. During the secretory-stage (left image), active Ca^2+^ transport on the lateral membrane is primarily mediated by PMCA1 and PMCA4 and ATP is hydrolyzed in the process. NCX1, NCX3, and NCKX3 mediate Ca^2+^ export in the Tomes' process. More calcium transporters are expressed during the maturation stage (right image). The CRAC channel, composed of the channel ORAI1 and the ER membrane calcium sensor STIM1, mediates the bulk of calcium entry and is active in response to ER calcium store depletion through IP_3_R. The cell then removes calcium from the cytoplasm through the SERCA2 pump that replenishes ER stores, and the NCKX and NCX proteins on the apical border plasma membrane that export calcium into the enamel area and facilitate mineralization (Nurbaeva et al., 2015a,b, 2017). NCX1 and PMCA4 export Ca^2+^ from the stratum intermedium (secretory-stage enamel organ) and papillary layer (maturation-stage enamel organ). Desmosomes (Sasaki et al., 1984; Fausser et al., 1998; Jheon et al., 2011) and gap junctions (Sasaki and Garant, 1986a,b,d; Pinero et al., 1994; Inai et al., 1997) have also been identified bridging or uniting adjacent epithelial cells in the enamel organ, and are also illustrated.

Our novel data adds to the current understanding of Ca^2+^ transport in secretory and maturation stage enamel organ, described in Figure 4. Ca^2+^ import by the CRAC channel, ER Ca^2+^ export by IP_3_R, and ER Ca^2+^ import by the SERCA2 pump have been well-described elsewhere (Nurbaeva et al., 2015a,b, 2017). In summary, when Ca^2+^ is released from the ER through IP_3_R, STIM1 associates with ORAI1 and forms the CRAC channel that allows Ca^2+^ to flow into the cell. Ca^2+^ is then removed from the cytoplasm through PMCA1 and PMCA4 on the basal and lateral membranes, NCX1, NCX3, NCKX3, and NCKX4 on the apical membrane, and SERCA2 on the ER membrane. NCX1 and PMCA4 are also involved in Ca^2+^ export in the stratum intermedium and papillary layer. This process of Ca^2+^ cycling occurs more during the maturation stage, when large amounts of Ca^2+^ are necessary for enamel mineralization. During the secretory stage, PMCA1, PMCA4, NCX1, NCX3, and NCKX3 are the known Ca^2+^ exporters expressed, but further studies are necessary to understand Ca^2+^ import and other mechanisms of Ca^2+^ export during enamel secretion.

## Conclusion

Based on the available data, we have reviewed and summarized the expression profiles of the major Ca^2+^ export pumps and exchangers in enamel organ cells (Table 3; Okumura et al., 2010; Hu et al., 2012; Wang et al., 2014), and illustrated these Ca^2+^ export proteins along with the current model for Ca^2+^ import in enamel organ cells as proposed by Nurbaeva et al. (2015a,b, 2017) (Figure 4). As future studies continue to better define Ca^2+^ export (and also Ca^2+^ import—see Nurbaeva et al., 2015a,b, 2017) during amelogenesis, the information in Table 3 will undoubtedly expand and become more precisely defined. Ultimately, elucidating the multitude of mechanisms involved in transcellular Ca^2+^ movements in the enamel-forming cells will result in a better understanding of the physiology and formation of enamel, the hardest and most calcified tissue in mammals.

**Table 3:** **Major and most highly expressed calcium export pumps and exchangers in enamel organ cells**.

**Gene/protein**	**Secretory ameloblasts**	**Stratum intermedium**	**Stellate reticulum**	**Maturation ameloblasts**	**Papillary layer cells**	**References**
Atp2b1/PMCA1	Basolateral	✓ (weak)	✗	Basolateral	✓ (Weak)	
Atp2b4/PMCA4	Basolateral	✓	✗	Basolateral	✓	
Slc8a1/NCX1	Apical	✓	✗	Apical	✓	Okumura et al., 2010
Slc8a3/NCX3	Apical	✗	✗	Apical	✗	Okumura et al., 2010
Slc24a3/NCKX3	Apical	✗	✗	Apical	✗	
Slc24a4/NCKX4	✗	✗	✗	Apical	✗	Hu et al., 2012; Wang et al., 2014

## Author contributions

SR, XW, CS, and MP designed the experiments and wrote the manuscript; SR and XW performed the experiments; SR, XW, KY, JC, CS, and MP analyzed the data; SR, XW, and MP prepared the figures and tables; All listed authors critically read, edited, and approved the final manuscript. MP accepts full responsibility for the integrity of the data analysis.

### Conflict of interest statement

The authors declare that the research was conducted in the absence of any commercial or financial relationships that could be construed as a potential conflict of interest.

## References

[B1] Arquitt C. K. Boyd C. Wright J. T. (2002). Cystic fibrosis transmembrane regulator gene (CFTR) is associated with abnormal enamel formation. J. Dent. Res. 81, 492–496. 10.1177/154405910208100712 12161463

[B2] Berridge M. J. Bootman M. D. Roderick H. L. (2003). Calcium signalling: dynamics, homeostasis and remodelling. Nat. Rev. Mol. Cell Biol. 4, 517–529. 10.1038/nrm1155 12838335

[B3] Bertini E. Des Portes V. Zanni G. Santorelli F. Dionisi-Vici C. Vicari S.. (2000). X-linked congenital ataxia: a clinical and genetic study. Am. J. Med. Genet. 92, 53–56. 10.1002/(SICI)1096-8628(20000501)92:1<53::AID-AJMG9>3.0.CO;2-F 10797423

[B4] Blair H. C. Robinson L. J. Huang C. L. Sun L. Friedman P. A. Schlesinger P. H.. (2011). Calcium and bone disease. Biofactors 37, 159–167. 10.1002/biof.143 21674636 PMC3608212

[B5] Borke J. L. Zaki A. E.-M. Eisenmann D. R. Mednieks M. I. (1995). Localization of plasma membrane Ca^2+^ pump mRNA and protein in human ameloblasts by *in situ* hybridization and immunohistochemistry. Connect. Tissue Res. 33, 139–144. 10.3109/03008209509016993 7554945

[B6] Bortolozzi M. Brini M. Parkinson N. Crispino G. Scimemi P. De Siati R. D.. (2010). The novel PMCA2 pump mutation Tommy impairs cytosolic calcium clearance in hair cells and links to deafness in mice. J. Biol. Chem. 285, 37693–37703. 10.1074/jbc.M110.170092 20826782 PMC2988374

[B7] Brini M. (2009). Plasma membrane Ca^2+^-ATPase: from a housekeeping function to a versatile signaling role. Pflugers Arch. 257, 657–664. 10.1007/s00424-008-0505-6 18548270

[B8] Brini M. Cali T. Ottolini D. Carafoli E. (2013). The plasma membrane calcium pump in health and disease. FEBS J. 280, 5385–5397. 10.1111/febs.12193 23413890

[B9] Brini M. Carafoli E. (2011). The plasma membrane Ca^2+^ ATPase and the plasma membrane sodium calcium exchanger cooperate in the regulation of cell calcium. Cold Spring Harb. Perspect. Biol. 3:a004168 10.1101/cshperspect.a004168 21421919 PMC3039526

[B10] Brini M. Carafoli E. Cali T. (2017). The plasma membrane calcium pumps: focus on the role in (neuro)pathology. Biochem. Biophys. Res. Commun. 483, 1116–1124. 10.1016/j.bbrc.2016.07.117 27480928

[B11] Bronckers A. Kalogeraki L. Jorna H. J. Wilke M. Bervoets T. J. Lyaruu D. M.. (2010). The cystic fibrosis transmembrane conductance regulator (CFTR) is expressed in maturation stage ameloblasts, odontoblasts and bone cells. Bone 46, 1188–1196. 10.1016/j.bone.2009.12.002 20004757 PMC2842452

[B12] Bronckers A. L. Lyaruu D. Jalali R. Medina J. F. Zandieh-Doulabi B. Denbesten P. K. (2015). Ameloblast modulation and transport of Cl^−^, Na^+^, and K^+^ during Amelogenesis. J. Dent. Res. 94, 1740–1747. 10.1177/0022034515606900 26403673 PMC4681480

[B13] Cai X. Lytton J. (2004). Molecular cloning of a sixth member of the K^+^-dependent Na^+^/Ca^2+^ exchanger gene family, NCKX6. J. Biol. Chem. 279, 5867–5876. 10.1074/jbc.M310908200 14625281

[B14] Curry M. C. Luk N. A. Kenny P. A. Roberts-Thomson S. J. Monteith G. R. (2012). Distinct regulation of cytoplasmic calcium signals and cell death pathways by different plasma membrane calcium ATPase isoforms in MDA-MB-231 breast cancer cells. J. Biol. Chem. 287, 28598–28608. 10.1074/jbc.M112.364737 22733819 PMC3436507

[B15] Curry M. C. Roberts-Thomson S. J. Monteith G. R. (2011). Plasma membrane calcium ATPases and cancer. Biofactors 37, 132–138. 10.1002/biof.146 21674637

[B16] Fausser J. L. Schlepp O. Aberdam D. Meneguzzi G. Ruch J. V. Lesot H. (1998). Localization of antigens associated with adherens junctions, desmosomes, and hemidesmosomes during murine molar morphogenesis. Differentiation 63, 1–11. 10.1046/j.1432-0436.1998.6310001.x 9615388

[B17] Giacomello M. De Mario A. Scarlatti C. Primerano S. Carafoli E. (2013). Plasma membrane calcium ATPases and related disorders. Int. J. Biochem. Cell Biol. 45, 753–762. 10.1016/j.biocel.2012.09.016 23041476

[B18] Hamada H. Petrino M. G. Kakunaga T. (1982). Molecular structure and evolutionary origin of human cardiac muscle actin gene. Proc. Natl. Acad. Sci. U.S.A. 79, 5901–5905. 10.1073/pnas.79.19.5901 6310553 PMC347018

[B19] Herzog C. R. Reid B. M. Seymen F. Koruyucu M. Tuna E. B. Simmer J. P.. (2015). Hypomaturation amelogenesis imperfecta caused by a novel SLC24A4 mutation. Oral Surg. Oral Med. Oral Pathol. Oral Radiol. 119, e77–e81. 10.1016/j.oooo.2014.09.003 25442250 PMC4291293

[B20] Ho P. W. Pang S. Y. Li M. Tse Z. H. Kung M. H. Sham P. C.. (2015). PMCA4 (ATP2B4) mutation in familial spastic paraplegia causes delay in intracellular calcium extrusion. Brain Behav. 5:e00321. 10.1002/brb3.321 25798335 PMC4356846

[B21] Hu J. C. Chun Y. H. Al Hazzazzi T. Simmer J. P. (2007). Enamel formation and amelogenesis imperfecta. Cells Tissues Organs 186, 78–85. 10.1159/000102683 17627121

[B22] Hu P. Lacruz R. S. Smith C. E. Smith S. M. Kurtz I. Paine M. L. (2012). Expression of the sodium/calcium/potassium exchanger, NCKX4, in ameloblasts. Cells Tissues Organs 196, 501–509. 10.1159/000337493 22677781 PMC3535175

[B23] Hubbard M. J. (1996). Articular debridement versus washout for degeneration of the medial femoral condyle. A five-year study. J. Bone Joint Surg. Br. 78, 217–219. 8666628

[B24] Hubbard M. J. (2000). Calcium transport across the dental enamel epithelium. Crit. Rev. Oral Biol. Med. 11, 437–466. 10.1177/10454411000110040401 11132765

[B25] Inai T. Nakamura K. Kurisu K. Shibata Y. (1997). Immunohistochemical localization of connexin43 in the enamel organ of the rat upper incisor during ameloblast development. Arch. Histol. Cytol. 60, 297–306. 10.1679/aohc.60.297 9376177

[B26] Jalloul A. H. Rogasevskaia T. P. Szerencsei R. T. Schnetkamp P. P. (2016a). A functional study of mutations in K^+^-dependent Na^+^-Ca^2+^ exchangers associated with amelogenesis imperfecta and non-syndromic Oculocutaneous Albinism. J. Biol. Chem. 291, 13113–13123. 10.1074/jbc.M116.728824 27129268 PMC4933227

[B27] Jalloul A. H. Szerencsei R. T. Schnetkamp P. P. (2016b). Cation dependencies and turnover rates of the human K^+^-dependent Na^+^-Ca^2^ exchangers NCKX1, NCKX2, NCKX3 and NCKX4. Cell Calcium 59, 1–11. 10.1016/j.ceca.2015.11.001 26631410

[B28] Jheon A. H. Mostowfi P. Snead M. L. Ihrie R. A. Sone E. Pramparo T.. (2011). PERP regulates enamel formation via effects on cell-cell adhesion and gene expression. J. Cell Sci. 124, 745–754. 10.1242/jcs.078071 21285247 PMC3039019

[B29] Josephsen K. Takano Y. Frische S. Praetorius J. Nielsen S. Aoba T.. (2010). Ion transporters in secretory and cyclically modulating ameloblasts: a new hypothesis for cellular control of preeruptive enamel maturation. Am. J. Physiol. Cell Physiol. 299, C1299–C1307. 10.1152/ajpcell.00218.2010 20844245

[B30] Khananshvili D. (2013). The SLC8 gene family of sodium-calcium exchangers (NCX) - structure, function, and regulation in health and disease. Mol. Aspects Med. 34, 220–235. 10.1016/j.mam.2012.07.003 23506867

[B31] Kim H. J. Prasad V. Hyung S. W. Lee Z. H. Lee S. W. Bhargava A.. (2012). Plasma membrane calcium ATPase regulates bone mass by fine-tuning osteoclast differentiation and survival. J. Cell Biol. 199, 1145–1158. 10.1083/jcb.201204067 23266958 PMC3529522

[B32] Krebs J. (2009). The influence of calcium signaling on the regulation of alternative splicing. Biochim. Biophys. Acta 1793, 979–984. 10.1016/j.bbamcr.2008.12.006 19133299

[B33] Lacruz R. S. Smith C. E. Bringasjr P. Chen Y. B. Smith S. M. Snead M. L.. (2012a). Identification of novel candidate genes involved in mineralization of dental enamel by genome-wide transcript profiling. J. Cell. Physiol. 227, 2264–2275. 10.1002/jcp.22965 21809343 PMC3243804

[B34] Lacruz R. S. Smith C. E. Kurtz I. Hubbard M. J. Paine M. L. (2013). New paradigms on the transport functions of maturation-stage ameloblasts. J. Dent. Res. 92, 122–129. 10.1177/0022034512470954 23242231 PMC3545694

[B35] Lacruz R. S. Smith C. E. Moffatt P. Chang E. H. Bromage T. G. Bringas P. Jr.. (2012b). Requirements for ion and solute transport, and pH regulation during enamel maturation. J. Cell. Physiol. 227, 1776–1785. 10.1002/jcp.22911 21732355 PMC3373187

[B36] Lee G. S. Choi K. C. Jeung E. B. (2009). K^+^-dependent Na^+^/Ca^2+^ exchanger 3 is involved in renal active calcium transport and is differentially expressed in the mouse kidney. Am. J. Physiol. Renal Physiol. 297, F371–F379. 10.1152/ajprenal.90615.2008 19474185

[B37] Lee K. H. Ho W. K. Lee S. H. (2013). Endocytosis of somatodendritic NCKX2 is regulated by Src family kinase-dependent tyrosine phosphorylation. Front. Cell. Neurosci. 7:14. 10.3389/fncel.2013.00014 23431067 PMC3576620

[B38] Lyaruu D. M. Bronckers A. L. Mulder L. Mardones P. Medina J. F. Kellokumpu S.. (2008). The anion exchanger Ae2 is required for enamel maturation in mouse teeth. Matrix Biol. 27, 119–127. 10.1016/j.matbio.2007.09.006 18042363 PMC2274829

[B39] Lytton J. (2007). Na^+^/Ca^2+^ exchangers: three mammalian gene families control Ca^2+^ transport. Biochem. J. 406, 365–382. 10.1042/BJ20070619 17716241

[B40] Mondal M. Sengupta M. Samanta S. Sil A. Ray K. (2012). Molecular basis of albinism in India: evaluation of seven potential candidate genes and some new findings. Gene 511, 470–474. 10.1016/j.gene.2012.09.012 23010199

[B41] Nurbaeva M. K. Eckstein M. Concepcion A. R. Smith C. E. Srikanth S. Paine M. L.. (2015a). Dental enamel cells express functional SOCE channels. Sci. Rep. 5:15803. 10.1038/srep15803 26515404 PMC4626795

[B42] Nurbaeva M. K. Eckstein M. Feske S. Lacruz R. S. (2017). Ca^2+^ transport and signalling in enamel cells. J. Physiol. 595, 3015–3039. 10.1113/JP272775 27510811 PMC5430215

[B43] Nurbaeva M. K. Eckstein M. Snead M. L. Feske S. Lacruz R. S. (2015b). Store-operated Ca^2+^ entry modulates the expression of enamel genes. J. Dent. Res. 94, 1471–1477. 10.1177/0022034515598144 26232387 PMC4577984

[B44] Okumura R. Shibukawa Y. Muramatsu T. Hashimoto S. Nakagawa K. Tazaki M.. (2010). Sodium-calcium exchangers in rat ameloblasts. J. Pharmacol. Sci. 112, 223–230. 10.1254/jphs.09267FP 20118617

[B45] Okunade G. W. Miller M. L. Pyne G. J. Sutliff R. L. O'connor K. T. Neumann J. C.. (2004). Targeted ablation of plasma membrane Ca^2+^-ATPase (PMCA) 1 and 4 indicates a major housekeeping function for PMCA1 and a critical role in hyperactivated sperm motility and male fertility for PMCA4. J. Biol. Chem. 279, 33742–33750. 10.1074/jbc.M404628200 15178683

[B46] Orrenius S. Gogvadze V. Zhivotovsky B. (2015). Calcium and mitochondria in the regulation of cell death. Biochem. Biophys. Res. Commun. 460, 72–81. 10.1016/j.bbrc.2015.01.137 25998735

[B47] Paine M. L. Wang H. J. Abuladze N. Liu W. Wall S. Kim Y. H. (2007). Expression of AE2 and NBC1 in secretory ameloblasts, in Experimental Biology Annual Meeting (Washington, DC).

[B48] Palmgren M. G. Nissen P. (2011). P-type ATPases. Annu. Rev. Biophys. 40, 243–266. 10.1146/annurev.biophys.093008.131331 21351879

[B49] Parry D. A. Poulter J. A. Logan C. V. Brookes S. J. Jafri H. Ferguson C. H.. (2013). Identification of mutations in SLC24A4, encoding a potassium-dependent sodium/calcium exchanger, as a cause of amelogenesis imperfecta. Am. J. Hum. Genet. 92, 307–312. 10.1016/j.ajhg.2013.01.003 23375655 PMC3567274

[B50] Penniston J. T. Padanyi R. Paszty K. Varga K. Hegedus L. Enyedi A. (2014). Apart from its known function, the plasma membrane Ca^2+^ ATPase can regulate Ca^2+^ signaling by controlling phosphatidylinositol 4,5-bisphosphate levels. J. Cell Sci. 127, 72–84. 10.1242/jcs.132548 24198396

[B51] Pinero G. J. Parker S. Rundus V. Hertzberg E. L. Minkoff R. (1994). Immunolocalization of connexin 43 in the tooth germ of the neonatal rat. Histochem. J. 26, 765–770. 10.1007/BF02388633 7883586

[B52] Riazuddin S. A. Shahzadi A. Zeitz C. Ahmed Z. M. Ayyagari R. Chavali V. R.. (2010). A mutation in SLC24A1 implicated in autosomal-recessive congenital stationary night blindness. Am. J. Hum. Genet. 87, 523–531. 10.1016/j.ajhg.2010.08.013 20850105 PMC2948789

[B53] Sasaki T. Garant P. R. (1986a). Fate of annular gap junctions in the papillary cells of the enamel organ in the rat incisor. Cell Tissue Res. 246, 523–530. 10.1007/BF00215192 3024840

[B54] Sasaki T. Garant P. R. (1986b). A study of post-secretory maturation ameloblasts in the cat by transmission and freeze-fracture electron-microscopy. Arch. Oral Biol. 31, 587–596. 10.1016/0003-9969(86)90082-8 3467683

[B55] Sasaki T. Garant P. R. (1986c). Ultracytochemical demonstration of ATP-dependent calcium pump in ameloblasts of rat incisor enamel organ. Calcif. Tissue Int. 39, 86–96. 10.1007/BF02553296 2943378

[B56] Sasaki T. Garant P. R. (1986d). An ultrastructural study of the papillary layer and its vascular bed in the kitten enamel organ. Anat. Rec. 214, 353–364. 10.1002/ar.1092140404 3518541

[B57] Sasaki T. Segawa K. Takiguchi R. Higashi S. (1984). Intercellular junctions in the cells of the human enamel organ as revealed by freeze-fracture. Arch. Oral Biol. 29, 275–286. 10.1016/0003-9969(84)90101-8 6586124

[B58] Schnetkamp P. P. (2004). The SLC24 Na^+^/Ca^2+^-K^+^ exchanger family: vision and beyond. Pflugers Arch. 447, 683–688. 10.1007/s00424-003-1069-0 14770312

[B59] Schuh K. Cartwright E. J. Jankevics E. Bundschu K. Liebermann J. Williams J. C.. (2004). Plasma membrane Ca^2+^ ATPase 4 is required for sperm motility and male fertility. J. Biol. Chem. 279, 28220–28226. 10.1074/jbc.M312599200 15078889

[B60] Seymen F. Lee K. E. Tran Le C. G. Yildirim M. Gencay K. Lee Z. H.. (2014). Exonal deletion of SLC24A4 causes hypomaturation amelogenesis imperfecta. J. Dent. Res. 93, 366–370. 10.1177/0022034514523786 24532815

[B61] Sharma V. O'halloran D. M. (2014). Recent structural and functional insights into the family of sodium calcium exchangers. Genesis 52, 93–109. 10.1002/dvg.22735 24376088

[B62] Shumilina E. Xuan N. T. Matzner N. Bhandaru M. Zemtsova I. M. Lang F. (2010). Regulation of calcium signaling in dendritic cells by 1,25-dihydroxyvitamin D3. FASEB J. 24, 1989–1996. 10.1096/fj.09-142265 20124438

[B63] Skobe Z. (2006). SEM evidence that one ameloblast secretes one keyhole-shaped enamel rod in monkey teeth. Eur. J. Oral Sci. 114, 338–342. 10.1111/j.1600-0722.2006.00305.x 16674709

[B64] Smith C. E. (1998). Cellular and chemical events during enamel maturation. Crit. Rev. Oral Biol. Med. 9, 128–161. 10.1177/10454411980090020101 9603233

[B65] Sokolow S. Manto M. Gailly P. Molgo J. Vandebrouck C. Vanderwinden J. M.. (2004). Impaired neuromuscular transmission and skeletal muscle fiber necrosis in mice lacking Na/Ca exchanger 3. J. Clin. Invest. 113, 265–273. 10.1172/JCI18688 14722618 PMC310749

[B66] Stephan A. B. Tobochnik S. Dibattista M. Wall C. M. Reisert J. Zhao H. (2011). The Na^+^/Ca^2+^ exchanger NCKX4 governs termination and adaptation of the mammalian olfactory response. Nat. Neurosci. 15, 131–137. 10.1038/nn.2943 22057188 PMC3245797

[B67] Street V. A. Mckee-Johnson J. W. Fonseca R. C. Tempel B. L. Noben-Trauth K. (1998). Mutations in a plasma membrane Ca^2+^-ATPase gene cause deafness in deafwaddler mice. Nat. Genet. 19, 390–394. 10.1038/1284 9697703

[B68] Strehler E. E. (2013). Plasma membrane calcium ATPases as novel candidates for therapeutic agent development. J. Pharm. Pharm. Sci. 16, 190–206. 10.18433/J3Z011 23958189 PMC3869240

[B69] Strehler E. E. Zacharias D. A. (2001). Role of alternative splicing in generating isoform diversity among plasma membrane calcium pumps. Physiol. Rev. 81, 21–50. 11152753 10.1152/physrev.2001.81.1.21

[B70] Takano Y. (1995). Enamel mineralization and the role of ameloblasts in calcium transport. Connect. Tissue Res. 33, 127–137. 10.3109/03008209509016992 7554944

[B71] Tsuchiya M. Sharma R. Tye C. E. Sugiyama T. Bartlett J. D. (2009). Transforming growth factor-beta1 expression is up-regulated in maturation-stage enamel organ and may induce ameloblast apoptosis. Eur. J. Oral Sci. 117, 105–112. 10.1111/j.1600-0722.2009.00612.x 19320718 PMC2711557

[B72] Vinberg F. Wang T. Molday R. S. Chen J. Kefalov V. J. (2015). A new mouse model for stationary night blindness with mutant Slc24a1 explains the pathophysiology of the associated human disease. Hum. Mol. Genet. 24, 5915–5929. 10.1093/hmg/ddv319 26246500 PMC4581614

[B73] Wakimoto K. Kobayashi K. Kuro O. M. Yao A. Iwamoto T. Yanaka N.. (2000). Targeted disruption of Na^+^/Ca^2+^ exchanger gene leads to cardiomyocyte apoptosis and defects in heartbeat. J. Biol. Chem. 275, 36991–36998. 10.1074/jbc.M004035200 10967099

[B74] Wang S. Choi M. Richardson A. S. Reid B. M. Seymen F. Yildirim M.. (2014). STIM1 and SLC24A4 are critical for enamel maturation. J. Dent. Res. 93, 94S–100S. 10.1177/0022034514527971 24621671 PMC4107542

[B75] Wei A. H. Zang D. J. Zhang Z. Liu X. Z. He X. Yang L.. (2013). Exome sequencing identifies SLC24A5 as a candidate gene for nonsyndromic oculocutaneous albinism. J. Invest. Dermatol. 133, 1834–1840. 10.1038/jid.2013.49 23364476

[B76] Wen X. Lacruz R. S. Smith C. E. Paine M. L. (2014). Gene-expression profile and localization of Na^+^/K^+^-ATPase in rat enamel organ cells. Eur. J. Oral Sci. 122, 21–26. 10.1111/eos.12106 24313748 PMC4005357

[B77] Yang H. Kim T. H. Lee H. H. Choi K. C. Jeung E. B. (2011). Distinct expression of the calcium exchangers, NCKX3 and NCX1, and their regulation by steroid in the human endometrium during the menstrual cycle. Reprod. Sci. 18, 577–585. 10.1177/1933719110396229 21321244

[B78] Yin K. Lei Y. Wen X. Lacruz R. S. Soleimani M. Kurtz I.. (2015). SLC26A gene family participate in ph regulation during enamel maturation. PLoS ONE 10:e0144703. 10.1371/journal.pone.0144703 26671068 PMC4679777

[B79] Zacharias D. A. Kappen C. (1999). Developmental expression of the four plasma membrane calcium ATPase (Pmca) genes in the mouse. Biochim. Biophys. Acta 1428, 397–405. 10.1016/S0304-4165(99)00058-6 10434059

[B80] Zaki A. E. Hand A. R. Mednieks M. I. Eisenmann D. R. Borke J. L. (1996). Quantitative immunocytochemistry of Ca^2+^-Mg^2+^ ATPase in ameloblasts associated with enamel secretion and maturation in the rat incisor. Adv. Dent. Res. 10, 245–251. 10.1177/08959374960100022101 9206344

[B81] Zanni G. Cali T. Kalscheuer V. M. Ottolini D. Barresi S. Lebrun N.. (2012). Mutation of plasma membrane Ca^2+^ ATPase isoform 3 in a family with X-linked congenital cerebellar ataxia impairs Ca^2+^ homeostasis. Proc. Natl. Acad. Sci. U.S.A. 109, 14514–14519. 10.1073/pnas.1207488109 22912398 PMC3437887

[B82] Zhekova H. Zhao C. Schnetkamp P. P. Noskov S. Y. (2016). Characterization of the cation binding sites in the NCKX2 Na^+^/Ca^2+^-K^+^ exchanger. Biochemistry 55, 6445–6455. 10.1021/acs.biochem.6b00591 27805378

