# Supplementary material for: Multiple Calcium Export Exchangers and Pumps Are a Prominent Feature of Enamel Organ Cells
Source: Front Physiol. 2017 May 23;8:336. doi: 10.3389/fphys.2017.00336 (PMC5440769; doi:10.3389/fphys.2017.00336)
Supplement: Supplementary file 1 [file Image1.PDF]

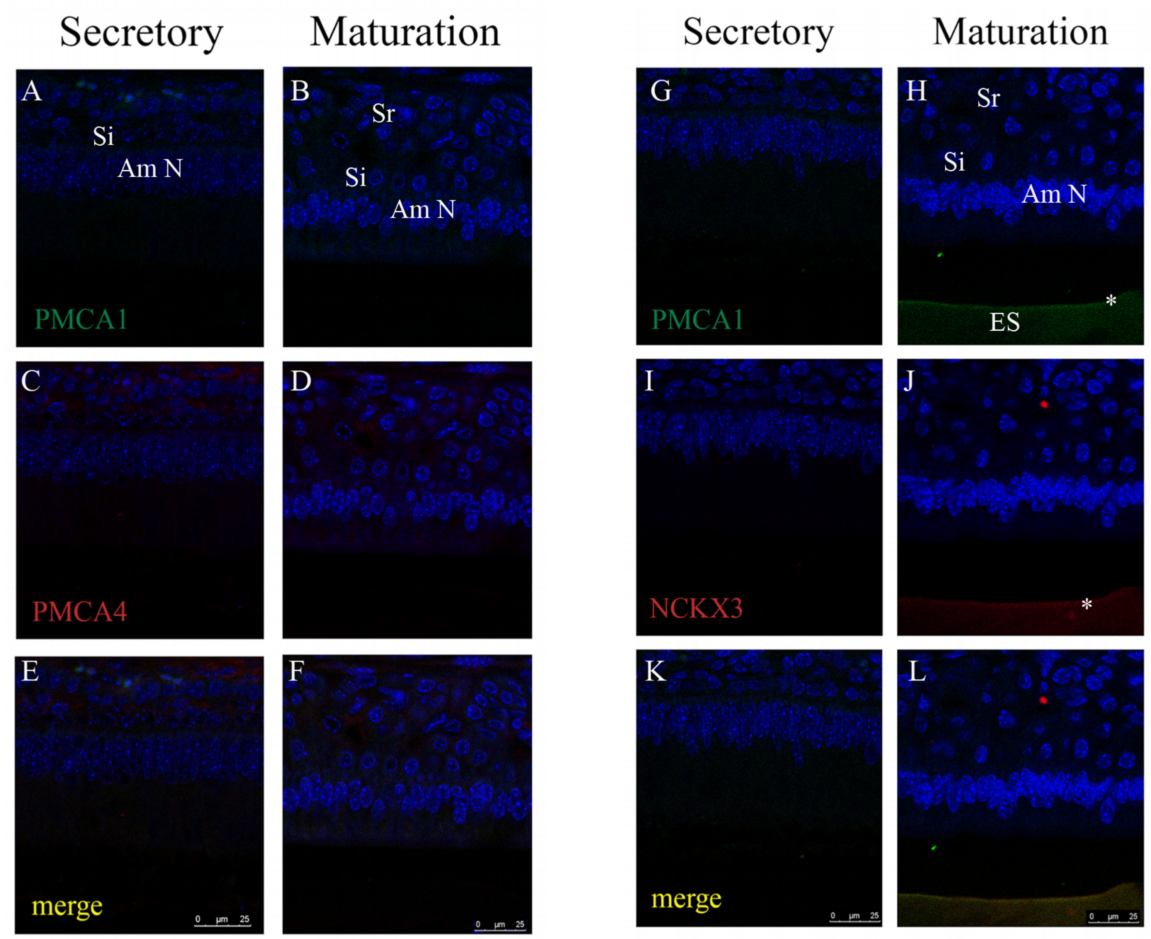

Supplemental Figure 1. No primary antibody controls for data presented in Figure 3. Columns from left to right show secretory stage (A, C, E, G, I, and K) and maturation stage (B, D, F, H, J, and L). Rows show immunoreactivity for the secondary antibodies used for PMCA1 (anti-rabbit, A, B, G and H), PMCA4 (anti-mouse, C and D) and NCKX3 (anti-goat, I and J). Merged images and scale bars are shown for each column (E, F, K, and L). Negligible autofluorescence is observed for any of the secondary antibodies described, under the same conditions as the slides used in Figure 3. For oridentation, using DAPI counter staining, ameloblast nuclei (Am N) are identified, as are nuclei of the cells of the stratum intermedium (Si) and stellate reticulum (Sr). The enamel space (ES) is also identified, and the union of the ameloblast apical membrane to the enamel space is identified with an asterisk (*).
